# The Effect of Static Stretch on Elastin Degradation in Arteries

**DOI:** 10.1371/journal.pone.0081951

**Published:** 2013-12-16

**Authors:** Ming-Jay Chow, Myunghwan Choi, Seok Hyun Yun, Yanhang Zhang

**Affiliations:** 1 Department of Mechanical Engineering, Boston University, Boston, Massachusetts, United States of America; 2 Department of Biomedical Engineering, Boston University, Boston, Massachusetts, United States of America; 3 Wellman Center for Photomedicine, Harvard Medical School and Massachusetts General Hospital, Cambridge, Massachusetts, United States of America; 4 Graduate School of Nanoscience and Technology (WCU), Korea Advanced Institute of Science and Technology, Daejeon, Korea; University of Akron, United States of America

## Abstract

Previously we have shown that gradual changes in the structure of elastin during an elastase treatment can lead to important transition stages in the mechanical behavior of arteries [1]. However, in vivo arteries are constantly being loaded due to systolic and diastolic pressures and so understanding the effects of loading on the enzymatic degradation of elastin in arteries is important. With biaxial tensile testing, we measured the mechanical behavior of porcine thoracic aortas digested with a mild solution of purified elastase (5 U/mL) in the presence of a static stretch. Arterial mechanical properties and biochemical composition were analyzed to assess the effects of mechanical stretch on elastin degradation. As elastin is being removed, the dimensions of the artery increase by more than 20% in both the longitude and circumference directions. Elastin assays indicate a faster rate of degradation when stretch was present during the digestion. A simple exponential decay fitting confirms the time constant for digestion with stretch (0.11±0.04 h^−1^) is almost twice that of digestion without stretch (0.069±0.028 h^−1^). The transition from J-shaped to S-shaped stress vs. strain behavior in the longitudinal direction generally occurs when elastin content is reduced by about 60%. Multiphoton image analysis confirms the removal/fragmentation of elastin and also shows that the collagen fibers are closely intertwined with the elastin lamellae in the medial layer. After removal of elastin, the collagen fibers are no longer constrained and become disordered. Release of amorphous elastin during the fragmentation of the lamellae layers is observed and provides insights into the process of elastin degradation. Overall this study reveals several interesting microstructural changes in the extracellular matrix that could explain the resulting mechanical behavior of arteries with elastin degradation.

## Introduction

The typical anisotropic and hyperelastic passive mechanical behavior of arteries can be attributed to the main structural components, elastin and collagen, in the extracellular matrix (ECM) [2], [3], [4]. In the stress vs. strain curve from a healthy artery, the elastic fibers support most of the load in the initial low sloped region [5]. The subsequent increase in stiffness is due to collagen fiber recruitment as coiled/wavy collagen fibers become straightened and start bearing the majority of load [6]. For a healthy artery exposed to diastolic and systolic pressures, both the elastin-supported and collagen-supported regions are encompassed [7]. The structure and composition of arteries can be dramatically altered as a result of changes in blood flow, pressure, and chemical factors [8], [9]. Significant arterial remodeling is seen in abdominal aortic aneurysm (AAA) where 90% reductions in elastin, indicators of excess aged collagen, and improper new collagen synthesis have been reported [10]. Fragmentation of the elastic laminae and fibers also occurs in the media layer [11]. As a result of these structural differences, human aortic aneurysm tissue has an increased elastic modulus and amount of anisotropy compared to healthy tissue [12], [13].

Chemical digestion models on animal tissue have often been used to mimic the elastin degradation caused by aneurysm because of the difficulties associated with obtaining human tissue [4]. Previously we used a mild solution of elastase paired with longer digestion times in order to record the gradual changes in mechanical properties and structure. As a result of elastin removal, the stress vs. strain curves progressed through four distinctly different mechanical stages [1]. Structural changes in adventitial collagen associated with elastin degradation were studied with multiphoton planar imaging and showed the collagen fibers are crimped before digestion and have reduced waviness after elastin removal [14].

In vivo arteries are constantly being stretched due to systolic and diastolic blood pressures. Earlier studies have shown that the enzymatic breakdown of the elastin and collagen ECM can be affected by the presence of static stresses [15], [16]. In vivo digestion methodologies have also been used to study the structural changes of arteries under enzymatic degradation with various models of AAA. Techniques can involve the topical application of elastase on exposed rabbit carotid arteries [17] or intraluminal delivery of the enzyme after temporarily blocking an artery with a balloon catheter or microvascular clamp [18], [19]. However, the effects of loading on the enzymatic degradation of elastin in arteries have not been studied.

Our focus here is to determine the biomechanical role of elastin in arteries and examine the effect of static stretch on the enzymatic degradation of arterial tissue. Porcine thoracic aortas are digested in vitro with mild elastase solutions while being stretched. Biaxial tensile testing is used to characterize the mechanical behavior and elastin assays are performed for quantification of biochemical changes in the ECM. Multiphoton microscopy is used to assess structural changes of elastic lamellae and ECM with elastin degradation.

## Materials and Methods

### Tissue preparation and elastin degradation

**Figure 1:**
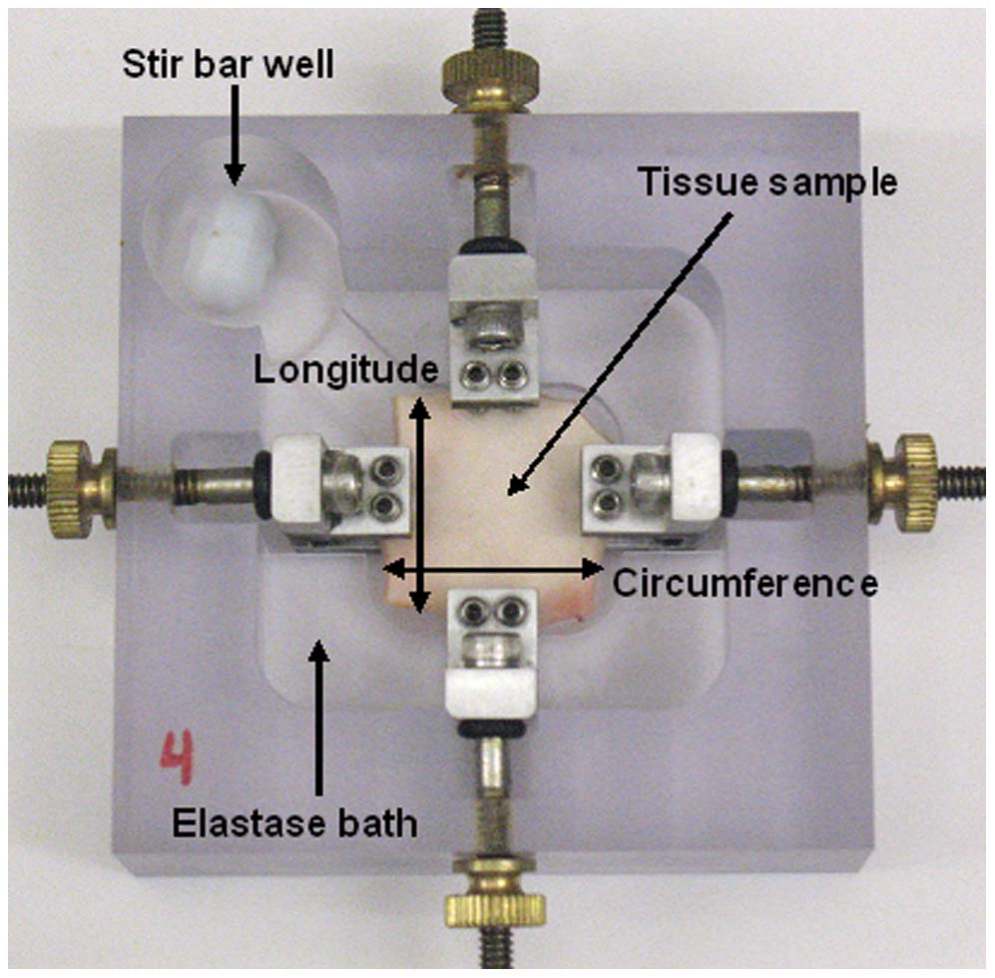
Tissue tension digestion bath that allows for static stretches to be applied to both the longitudinal and circumferential directions of the sample during enzymatic degradation.

Porcine thoracic aortas of 12–24 months old pigs were obtained from a local abattoir (Research 97 Inc, MA) with permission to use the tissue in this study. Approximately 20 mm sized square samples were cut so that one edge was parallel to the longitudinal direction and the other edge was parallel to the circumferential direction of the artery. Samples of similar thicknesses were placed in 1× phosphate buffered saline (PBS) solution at 4°C and all samples were mechanically tested at the fresh condition within 24 hours of tissue harvesting. A 5 U/mL ultra-pure elastase solution (MP Biomedicals, LLC, OH) at 37°C was used to gradually degrade tissues [20]. A total of 68 samples were tested at six time groups (6, 7.5, 9, 12, 24, and 48 hours, with n = 12 at all time points except n = 14 at 6 hours and n = 6 at 48 hours). The experimental time points were weighted towards the shorter digestions in order to capture the transition from J-shaped to S-shaped stress-strain behavior. During elastin digestion, samples were placed in individual biaxial stretching devices for digestion (Figure 1) and extended to a stretch ratio of 1.25 with respect to the starting length in both the longitudinal and circumferential directions. These stretch ratios were representative of the stretch experienced by porcine arteries under physiological pressures according to previous literature [21], [22]. Following the end of the digestion, samples were rinsed to remove excess elastase and post-digestion mechanical testing was immediately performed.

### Mechanical testing

The experimental settings for the biaxial tensile testing and elastin assays are provided in greater detail in our previous study [1]. Briefly, equibiaxial tension tests were performed using a biaxial tensile tester controlled with a custom Labview program. Load cells captured the applied load and the position of marker dots was tracked with a CCD camera to measure the stretch in the two directions. For all mechanical tests, a small preload of 2±0.050 N/m was applied in order to straighten the sutures connecting the tissue to the device. Initially the samples were subjected to a series of eight preconditioning cycles in which they were loaded in both directions to 30 N/m for all tissues. Following preconditioning, eight cycles of equi-biaxial tension was applied to capture the anisotropic mechanical behavior. Cauchy stresses were calculated based on plane stress and incompressibility assumptions [5]:
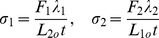
(1)


In Equation (1), σ is the Cauchy stress, *F* is the applied load, λ is the stretch, *L*_o_ is the initial length, and *t* is the thickness of the tissue. The subscripts 1 and 2 correspond to the longitudinal and circumferential directions of the tissue, respectively. The resulted stresses generally exceed 120 kPa in order to capture as much of the artery behavior as possible within the physiological pressure range. Cauchy stress vs. Green strain was plotted to describe the mechanical response. Green strain *E* was calculated as shown in equation (2) where λ is the stretch and *i* = 1, 2 [23].
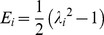
(2)


### Elastin assay

After mechanical testing, tissues were sliced into smaller pieces for collagen and elastin assays. Following manufacture's protocols for the isolation of elastin using progressive treatments with 0.25M oxalic acid, the Fastin elastin assay kit (Biocolor, www.biocolor.co.uk) measured soluble tropoelastins and α-elastin polypeptides by first binding a dye label (5,10,15,20-tetraphenyl-21H,23H-porphine tetra-sulfonate) to the basic and non-polar amino acid sequences in mammalian elastin. The optical densities of the labeled samples were then measured at 513 nm using a SpectraMax M5 plate reader (Molecular Devices) and compared to a control ladder to determine the mass fraction of elastin in tissue samples. Elastin content was expressed as µg of elastin/mg of wet tissue weight.

### Multiphoton imaging and analysis

Tissue samples from each group were frozen, cut into ∼200 µm thick cross sectional slices, and placed on slides. A custom-made two-photon laser scanning microscope with a tunable Ti:Sapphire laser (Mai-Tai DeepSee, Spectra-Physics) and a water immersion objective lens (20X, 1.0 NA) were used. In order to examine the continuity of the lamellae sheets in the medial layer during degradation, the circumferential cross section of the tissue was analyzed. The excitation wavelength was set to 810 nm and power was tuned to approximately 25 mW at the sample. A second harmonic signal (SHG) was generated and emissions detected at 405±10 nm to image collagen while the two-photon excited fluorescence (2PEF) was collected at 525±25 nm to image elastin.

The lengths of the elastic lamellae were quantified with a plug-in for ImageJ called NeuronJ [24]. The software allows the user to trace elongated image structures and has been successfully used in examining the waviness of adventitial collagen fibers [25], [14]. From the plug-in, the end-to-end distance *l*, and total fiber length, L, can be measured.

### Statistical analysis

Experimental data were summarized with mean ± standard deviations (SD). Values of elastin in arteries digested with and without stretch were compared using a factorial analysis of variance. The same statistical methods were used to compare the longitudinal and circumferential tissue size and lamellae length of the digested samples to the corresponding measurements taken from fresh tissue. A two-tailed *P*<0.05 was considered statistically significant with post-hoc testing using the Tukey's method to adjust for multiple comparisons. Statistical analysis was performed using the JMP statistical package (version 9.0.2, 2010 SAS Institute Inc.).

## Results

**Figure 2:**
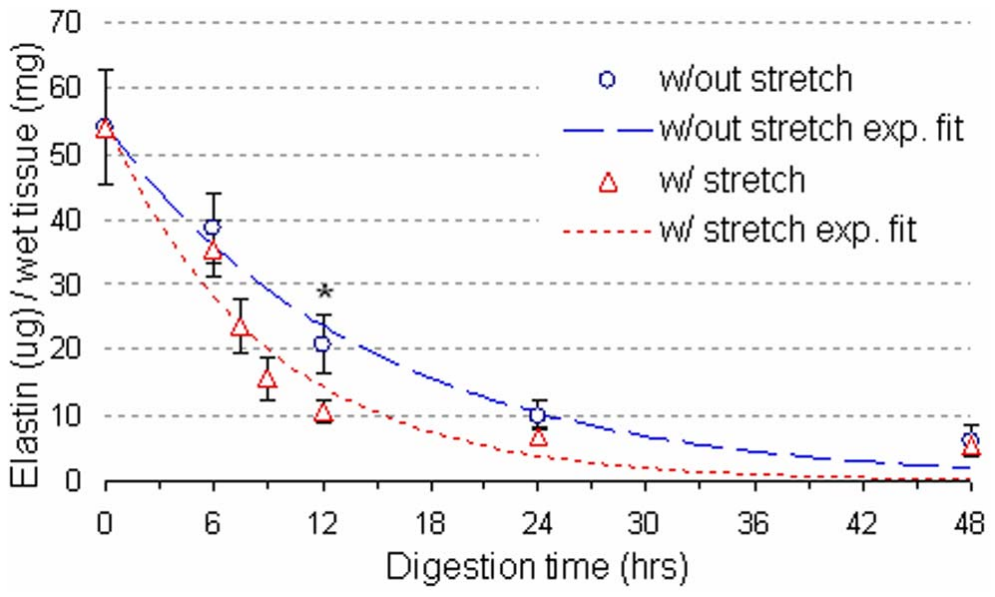
Elastin content of arterial tissue after digestion with stretch (triangles) and without stretch (circles). Lines indicate simple exponential fits. For comparisons between digestions with and without stretch, ^*^ P<0.05.

Figure 2 shows the elastin content in the tissue sample vs. digestion time. Fresh arteries have elastin content of 56.5±8.8 µg elastin/mg wet tissue weight and as expected, the elastin content continuously decreases with exposure to elastase. The rate of digestion was fitted with a simple exponential decay (Equation 3) and results from our previous work on elastin digestion without stretch [1] are also shown. The coefficients, *a*, were similar for digestions with and without tension at 57±12 and 52±7 (µg elastin/mg wet tissue weight) respectively. However, the time constant, *b*, for the digestion with stretch was −0.11±0.04 h^−1^ (w/ 95% CI) while the time constant for digestion without stretch was −0.069±0.028 h^−1^ (w/ 95% CI), indicating a faster rate of digestion when stretch is applied to the tissue.
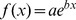
(3)


**Figure 3:**
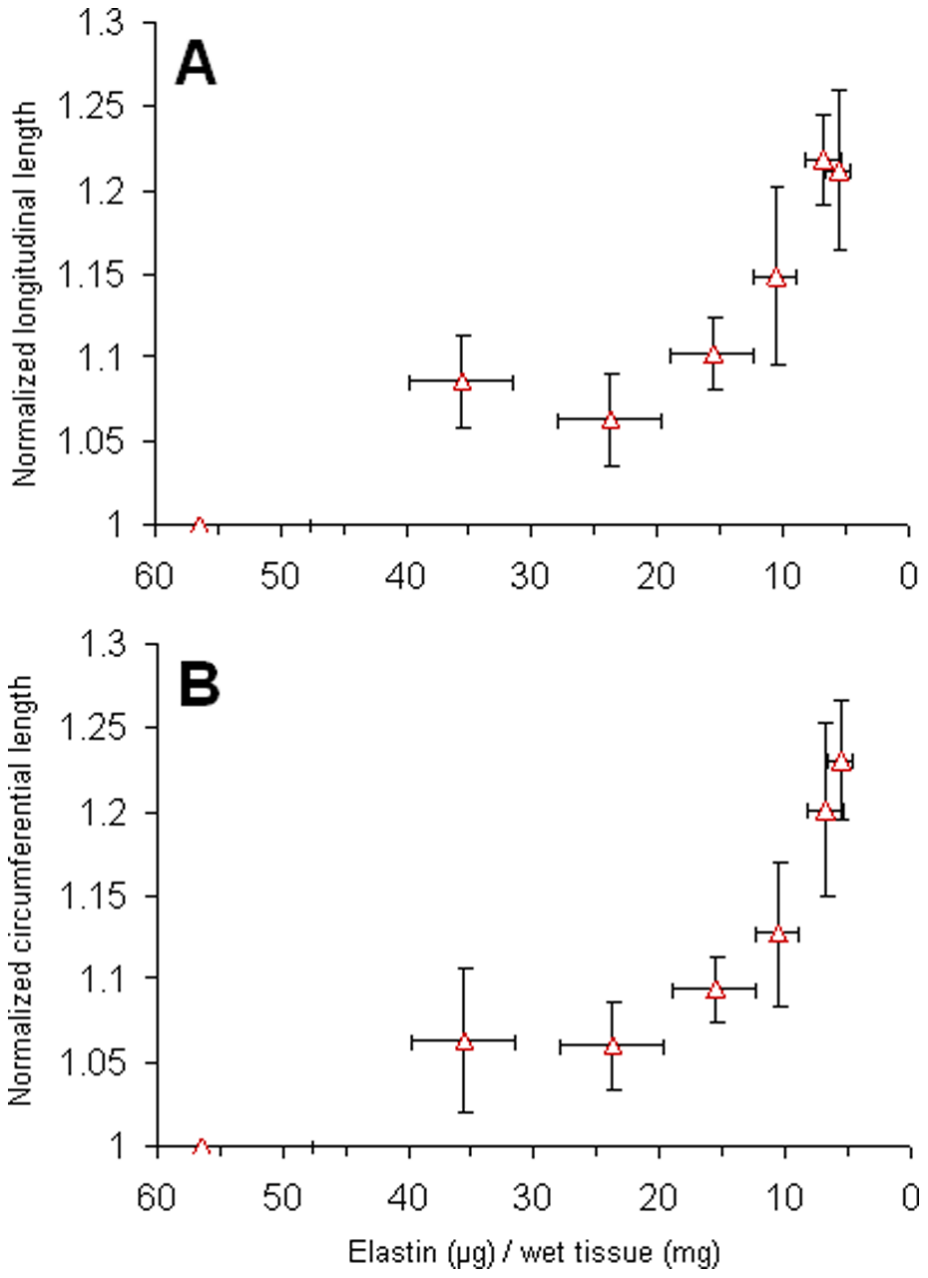
Changes in the size of the longitudinal (A) and circumferential (B) directions of the tissue after treating with elastase with stretch. The lengths of post-digestion samples were normalized to their individual fresh tissue measurements. All digestion groups were significantly greater than the fresh condition, P<0.05.

Figures 3A and 3B show the normalized longitudinal and circumferential dimensions of the tissues digested with stretch plotted against the elastin content. The in plane tissue dimensions increased by 20.1±5.1% and 21.8±2.7% in the circumferential and longitudinal directions respectively when minimal amount of elastin remained in the tissue.

**Figure 4:**
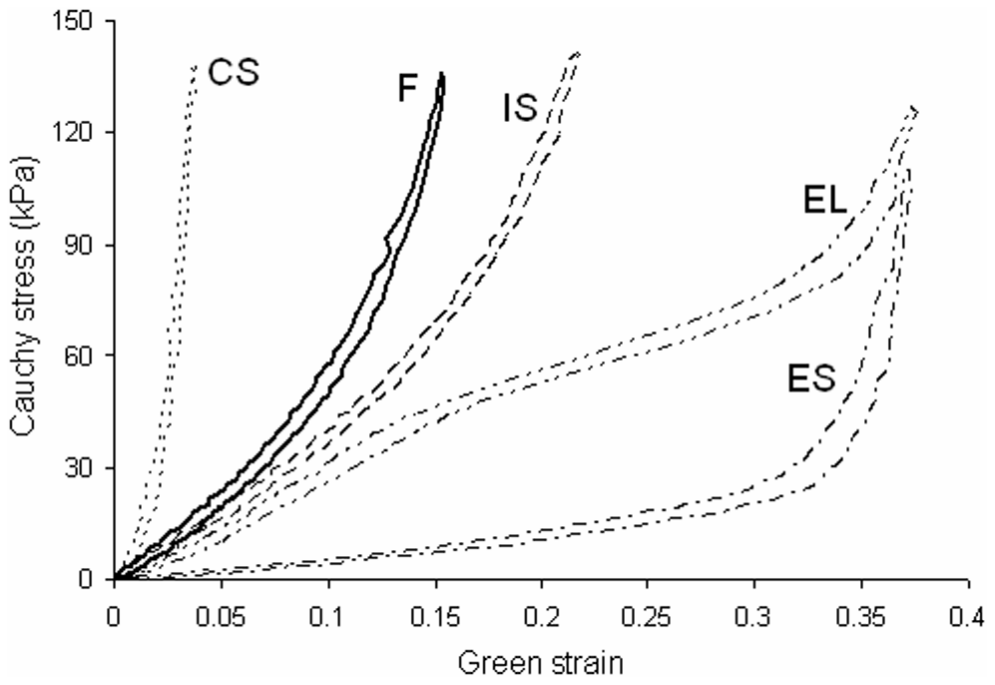
Representative Cauchy stress vs. Green strain curves from the longitudinal direction showing four major stages of the mechanical properties: initial-softening (IS), elastomer-like (EL), extensible-but-stiff (ES), and collagen-scaffold (CS) behavior. The behavior of fresh (F) arteries is also shown for comparison.

Figure 4 shows the representative mechanical behavior of a fresh artery and the four types of stress vs. strain curves as a result of digestion. Only the longitudinal direction is displayed in Figure 4. Fresh arteries generally have anisotropic and hyperelastic behavior with the circumferential direction being stiffer than the longitudinal direction. As a result of the degradation process, the mechanical behavior progressed through the four mechanical stages discussed before [1]. In the initial-softening (IS) stage, the initial slopes of the curves are mildly reduced, but the overall shape is still anisotropic and hyperelastic. In the elastomer-like (EL) stage, the stress vs. strain curve loses the J-shape and becomes more S-shaped. When little elastin remains in the extensible-but-stiff (ES) stage, the mechanical behavior is characterized by a pronounced J-shape curve with a very low initial slope, extended toe region, and prominent strain stiffening. Finally when the ECM has been reduced to essentially a collagen scaffold, the arteries are very stiff at the onset of loading with no toe region which is termed the collagen-scaffold (CS) stage.

**Figure 5:**
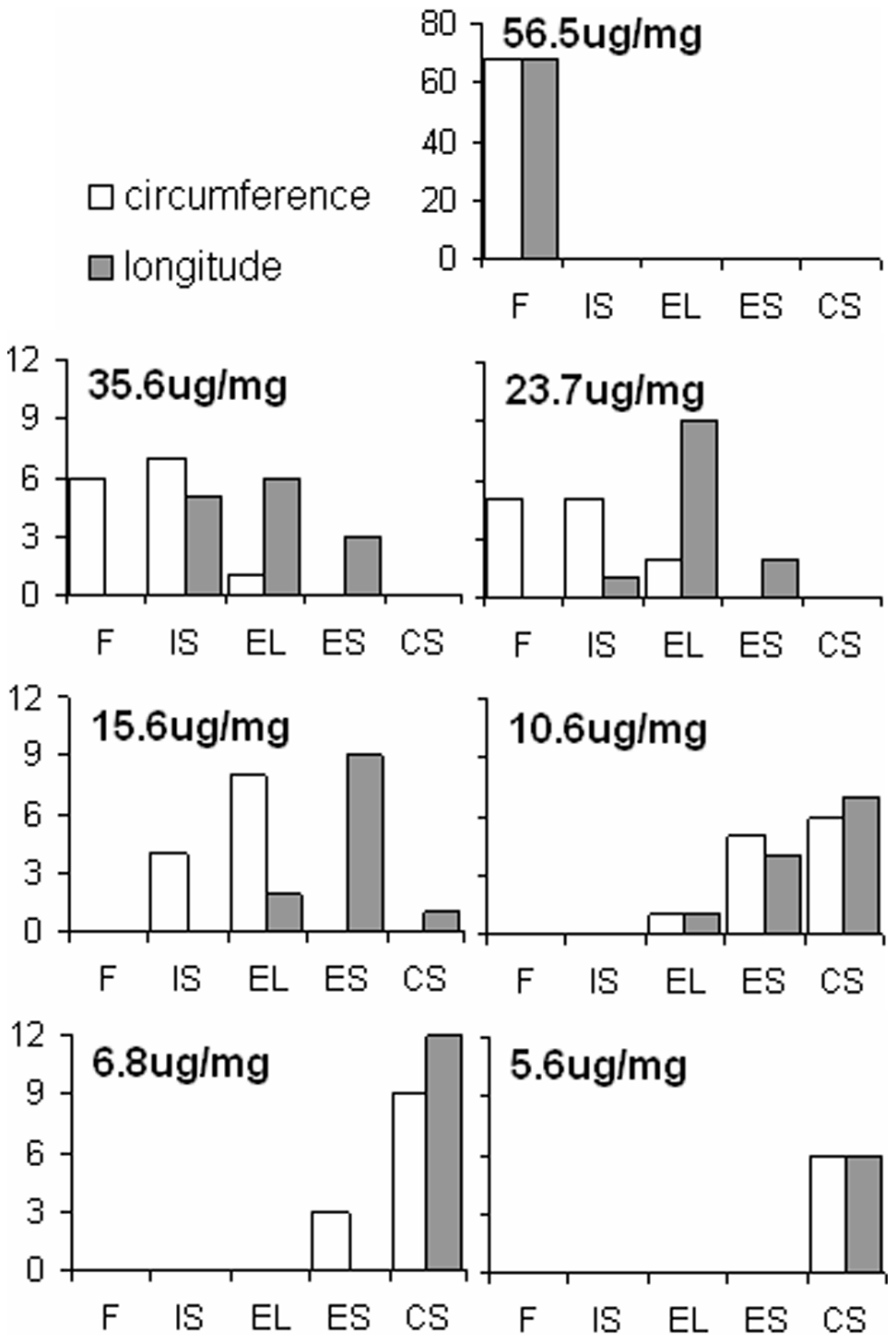
Distribution of arteries after grouping the stress vs. strain behavior of digested samples into one of the four mechanical stages shown in Figure 4 after digestion with static stretch. The circumferential and longitudinal behavior appears as white and grey columns, respectively.

To better understand the transition of mechanical stages during elastin degradation, Figure 5 shows the distribution of arteries after grouping the stress vs. strain behavior of digested samples into one of the four mechanical stages. The groups are labeled with the amount of elastin and once again fresh arteries had an average elastin content of 56.5±8.8 µg elastin/mg wet tissue weight. The distributions show the longitudinal direction progresses more quickly through the four stages compared to the circumferential direction. The transition from the J-shaped to S-shaped stress-strain behavior occurs in the longitudinal direction when the elastin content was 23.7±4.1 µg elastin/mg wet tissue. The transition occurred in the circumferential direction when the elastin content was 15.6±3.2 µg elastin/mg wet tissue weight.

**Figure 6:**
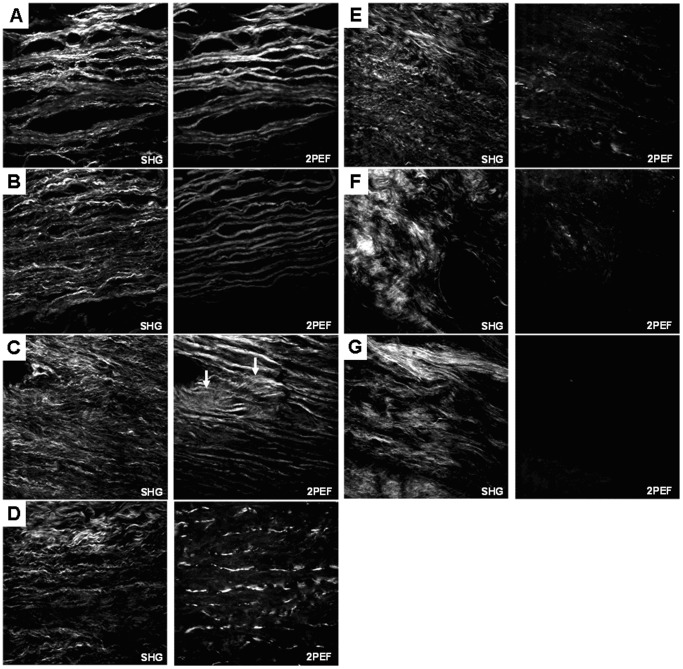
Multiphoton images of cross sections of arteries after digestion with static stretch with 56.5, 35.6, 23.7, 15.6, 10.6, 6.8, and 5.6 µg elastin/mg wet tissue (A through G respectively) with the SHG signal (collagen, shown on left) and 2PEF signal (elastin, shown to the right) separated from a single image. There is increased fragmentation of elastic lamellae layers and collagen fibers become more disorganized with elastin removal. Images are 275×275 µm.

**Figure 7:**
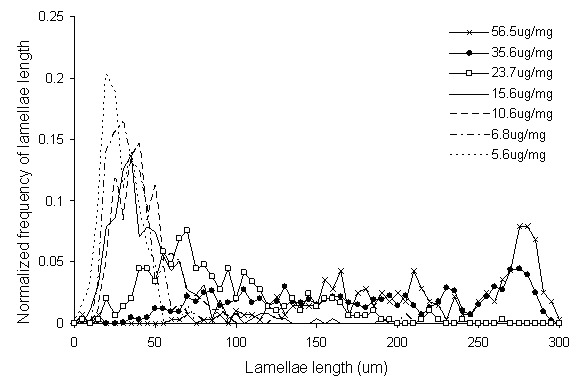
Normalized frequency vs. elastic lamellae length in arterial tissue after digestion with static stretch. Evidence of increased fragmentation is shown as distribution of lamellae lengths shifts from right (longer) to left (short fragments). All groups have a significantly decreased mean length compared to the undigested arteries, P<0.05.

In Figure 6, multiphoton cross sectional images of samples at various elastin contents are shown with the collagen in the left column and the elastin in the right. Qualitatively, the images show that the concentric elastic lamellae layers become fragmented as a result of the elastase degradation. The collagen fibers in the medial layer are much smaller in diameter and appear to be arranged parallel to the elastic lamellae structure. As the lamellae are degraded, the collagen fibers become disorganized and eventually form clumps of straightened relaxed fibers. Due to the collagen fibers being much finer and denser in the medial layer, only the length of the elastic lamellae are measurable with the NeuronJ plugin. In Figure 7, the normalized frequencies of lengths of the elastic lamellae are shown. The normalized frequency was calculated by dividing the number of lamellae at a certain length by the total number of lamellae that were measured in that group. When the tissue is undigested, lamellae lengths are longer with lengths widely distributed from 150–300 µm. As the elastin content is reduced, the lamellae lengths are significantly shortened and the distribution of lengths becomes narrower. Finally when there is a minimum amount of elastin in the arteries, the lamellae length distribution is captured by a single peak between 0–50 µm.

## Discussion

This study examined the effect of static stretch on the enzymatic degradation of elastin in arterial tissue. The increased rate of degradation of elastin with the presence of loading has been shown previously in elastin rich cell sheets [15] and was confirmed by this study (Figure 2). While the initial and final elastin content was similar for digestions with and without stretch, the presence of loading during digestion decreased the amount of time necessary to reach the final elastin depleted state. Previous studies have investigated the mechanisms for the faster elastin degradation under loading. Their results suggest that the mechanical stress on tissue could either make more binding sites available to the elastase enzyme or change the binding kinetics to allow the enzyme to cleave elastin more easily [26], [27].

**Figure 8:**
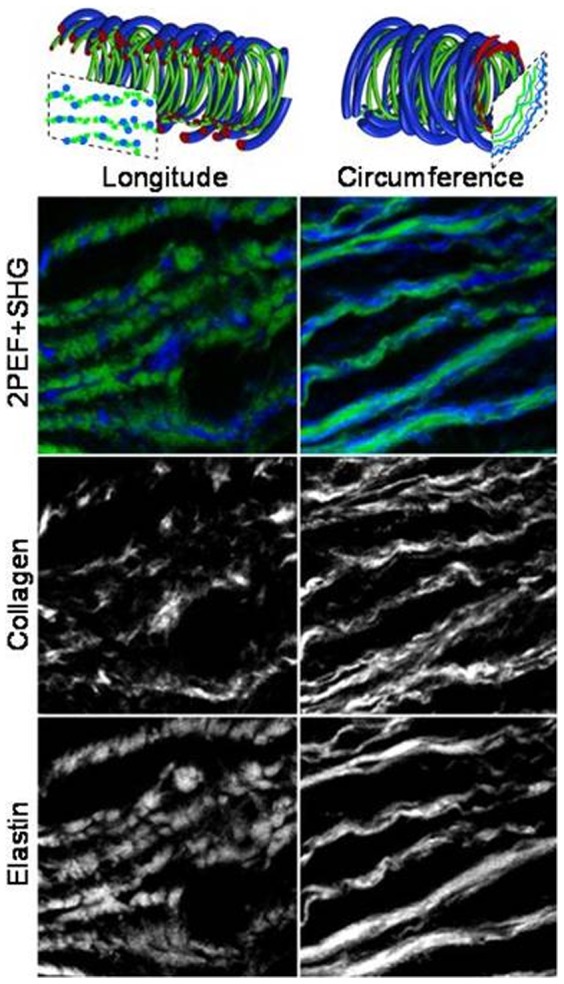
Schematic diagram of the expected appearance of the collagen (blue) and elastin (green) fibers after cross sectioning the artery in the longitudinal (left) and circumferential (right) directions. 2PEF and SHG images are cropped to 80×80 µm.

Evidence that the presence of tension during digestion increases the rate of degradation of elastin was also seen from mechanical testing results. Similar to the results from Chow et al. [1], the arteries progressed through the same four mechanical stages but went through them more quickly when digested with stretch. The elastomer-like stage still occurred when elastin content was reduced to 29–45% of the original amount (Figure 5). The fact that the transition from J-shaped to S-shaped stress-strain behavior is still present in tissues digested with stretch is important because it suggests rapid dilation due to ECM remodeling could also happen in vivo and may play a role in the formation and growth of aneurysm. It is also interesting that the longitudinal direction progresses through the four stages faster than the circumferential direction. This could be due to the preferred circumferential orientation of the collagen fibers, which may assist in maintaining the artery structure and resisting dilation due to elastin degradation. As shown in Figure 8, the ECM fibers appear more fragmented in the images of longitudinally cut tissue compared to those in the circumferentially cut tissue. Because there are fewer longitudinally oriented collagen fibers, the removal of elastin will affect that direction of the tissue to a greater extent leading to the faster progression through the four mechanical stages.

The general fragmentation of the elastic lamellae shown by the multiphoton images (Figure 6) and quantified with NeuronJ (Figure 7) concurs with other studies that also report this characteristic in aneurysm [28], [29]. When elastin is relatively unaltered, the lamellae layers (Figures 6A, 6B) extend from left to right across the entire image in solid lines. When the elastin content was reduced to 15.6±3.2 and 23.7±4.1 µg elastin/mg wet tissue (Figures 6C, 6D), the lamellae are fragmented and extend across the image in dashed lines suggesting the lamellae layers retained their overall end-to-end shape while degradation occurs. This is an indication that the collagen network is intertwined with the elastic lamellae and the interaction between the two components helps to maintain the end to end shape of the lamellae. Others have shown that collagen fibers provide resistance to the coiling of elastin as arteries treated with collagenase were reported to have a reduction of tissue dimensions [30], [31].

The loss of organization and straightening of collagen fibers with extensive elastin removal (Figure 6G) has been shown previously with planar multiphoton imaging of the adventitial collagen [14]. The cause of this loss of structure in the remaining collagen could be that porcine pancreatic elastase not only removes elastin but also degrades proteoglycans (PGs) in the ECM [32], [33]. PGs surround and support the elastin and collagen matrix [34] and smaller PGs like decorin can even bind to collagen to regulate cross-linking [35]. After selectively removing PGs from rat mesenteric arteries, the tissues are less capable of maintaining their shape which indicates the PGs are important to the structural integrity of the ECM [36]. Similar losses of undulation of collagen fibers and overall arterial geometry are reported from previous elastase treatment studies [37], [30], [31].

**Figure 9:**
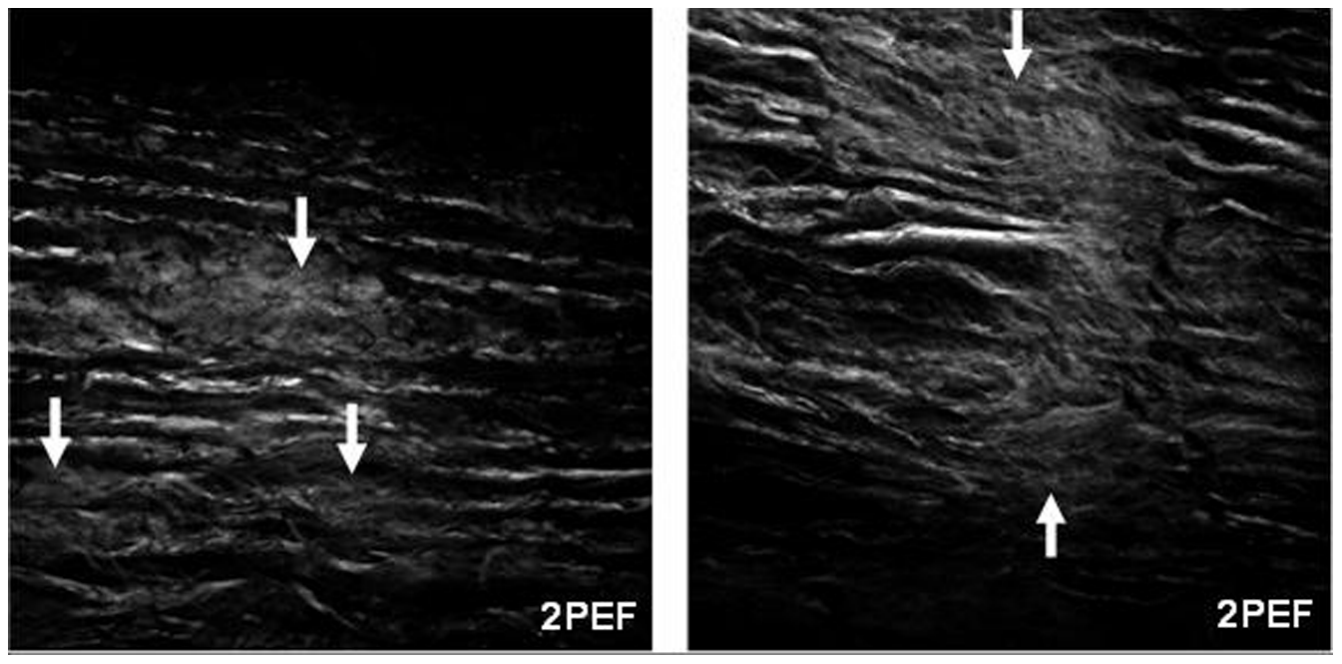
Multiphoton 2PEF images of samples showing regions where the boundaries of the elastic lamellae layers are compromised and loose amorphous elastin has been exposed as a result of elastase digestion. Images are 275×275 µm.

Multiphoton imaging reveals several interesting structural changes including: the release of amorphous elastin, the fragmentation of the elastic lamellae, and the formation of sandwiched layers. These microstructural alterations would cause changes in tissue mechanical properties. Elastic fibers have a structure that consists of amorphous elastin surrounded by a microfibril sheath [38], [39], [40]. As seen in the 2PEF imaging, large clumps of tangled amorphous elastin appear within the fragmented regions of the elastic lamellae due to elastin degradation (Figure 6C and 9). Because porcine pancreatic elastase also breaks down microfibrils, the elastase digestion seems to cause amorphous elastin to be released from their confined space within the lamellae sheets. The elastic lamellae have been described as elastic reservoirs that serve to distribute loading through the vessel wall [41], [39], [42]. The combination of fragmented lamellae and local pockets of amorphous elastin could lead to uneven load distribution and the immediate recruitment of stiffer collagen fibers. In addition, a feature of the in vitro elastin degradation in this study was that the digestion of the elastin starts from the exterior faces and progresses towards the center of the media. The complex sandwich structure may also alter the load distribution of the artery and contribute to the development of S-shaped stress-strain behavior.

## Limitations

There are several limitations in this study and improvements could be made to better understand the role of elastin/collagen as structural components of the ECM. While we have investigated the effect of static stretch on the digestion process, physiological loading is much more complex due to cyclic diastolic and systolic pressures as well as the formation of different blood flows in the dilated tissue [43]. Such limitations could be overcome by loading the tissue in a bioreactor, or incorporating an animal model with the controlled gradual elastin degradation. Because of the size of the multiphoton images, the maximum measurable length was limited and elastic lamellae certainly extend beyond the 300 µm length measured in our images. Another limitation is we were able to quantify elastic lamellae length and waviness in the media, but were unable to investigate potential changes in the collagen structure in the cross sectional images. The type III collagen fibers in the medial layer are of a much smaller diameter compared to the elastic lamellae and do not generate a strong SHG signal. The use of collagen specific fluorescent agents could be used to enhance the imaging of the collagen fibers. The fiber-level structural changes are also better revealed by in-plane images, as shown in our previous study [14].

## Conclusions

This study examines the effects of static stretch on the degradation of elastin in arteries. Our results show that the presence of mechanical stretch during elastase digestion increases the rate of degradation but does not affect the total amount of artery dilation or the final composition of the ECM. The arteries still progressed through the four mechanical stages when the gradual elastin degradation occurred with stretch. The transition from J-shaped to S-shaped behavior has the potential to allow for large changes in artery dimensions and may contribute to dilation during aneurysm formation. Multiphoton images reveal several interesting structural changes as a result of elastin degradation. Changes such as the fragmentation of the lamellae, release of amorphous elastin, and eventual loss of integrity of remaining collagen could be the potential causes of the different mechanical behavior.
